# Buffalo State Asylum for the Insane

**Published:** 1888-03

**Authors:** 


### Editorial.

---

### *BUFFALO STATE ASYLUM FOR THE INSANE.*

We are in receipt of the seventeenth annual report of this institution, although it is but the seventh since the Asylum was opened for the reception of patients. The managers state that the institution is in excellent financial condition, and that in the medical treatment, and in provisions for the comfort and care of patients, the Asylum has kept abreast with the advances in similar institutions, both in this country and abroad. This is certainly mild praise, for few institutions have that provision for comfort that this Asylum affords, and in the medical treatment, Superintendent Andrews and his corps have not only kept abreast with medical progress of the age, but have furnished much for the advancement of this special practice. They further report that the past year has been prolific in attacks upon asylums and their management. The public press has devoted columns to the sensational recital of alleged abuses, and charged serious crimes against the authorities and employees of institutions for the insane. In some cases, the occasion has been found in the accidents incident to the care of violent and dangerous lunatics. This Asylum has been subjected to a most unjust attack, based upon a death which occurred within its walls. The case is well known to our readers, and we need only to congratulate the Asylum and the accused attendants on their complete vindication. Although the Asylum brought the attention of the authorities to this accident, and asked for a judicial inquiry, the final attitude of certain daily papers in this vicinity made it appear that the whole management of the institution was under investigation. Our congratulations are, therefore, *apropos* on the vindication of the Asylum management, for it was shown to be complete in all its details.

The Superintendent's report contains many interesting facts. The statistics of admission and discharge for the seven years since the Asylum was opened, are as follows :

There have been admitted 1,969 patients, and 1,613 discharged; of these, 815 were recovered and improved, showing that fifty per cent. were benefited by treatment. By taking out the deaths and those discharged as not insane, mostly inebriates, the percentage is raised to more than sixty-one. These figures must be most gratifying to the medical staff, and are a source of just pride and pleasure to the physicians in the community who send patients to the institution. Many times physicians see the urgent necessity of sending patients to an asylum, but the friends object, as it seems to them but a place for safe care and keeping, but when we are able to hold up to them the prospect of recovery in so great a per cent. of cases as this, we certainly can do so with a clear and definite idea of our duty in the matter, and with a prospect of the endorsement of the patient himself when restored to reason.

The next point in the report is the small number admitted for the second time. Out of 297, but thirty-five were admitted during the second attack, five during the third, and two during the fourth. The condition in which they were received is also very interesting. The addition of a statement concerning these individual cases after treatment would have proved instructive.

Forty-three of those admitted were emaciated and feeble, eleven had heart disease in some of its various forms, nine had paralysis, three had fracture of skull, one, fracture of a rib, eight had bruises, cut throats, or other evidences of suicidal attempts, one was deaf and dumb, one had chorea, two had lost an eye, one an arm, four had acute meningitis, one had rickets, one, ovarian tumor, and one, heart disease, locomotor-ataxia and paraplegia.

Of the 318 patients admitted, 119 manifested suicidal or homicidal tendencies. There were admitted during the year 318, and discharged, 360. This is certainly very encouraging, and indicates that none are detained beyond the proper time.

